# Obituary

**Published:** 1853-07

**Authors:** 


*Obituary.*—In compliance with the request of the Livingston Co. Med. Society, we insert the following resolutions. With the late Dr. Dwight we were long acquainted. We knew him as a medical student more than a quarter of a century ago. He was a man of sterling worth, professional, social and moral. We bade him adieu at the meeting of the American Medical Association the evening before the terrible accident at Norwalk, of which he was one of the victims. He promised, at parting, to send, as a contribution to this Journal, the paper communicated by him to the association on the subject of chloroform. In the place thereof, it becomes our melancholy duty to insert resolutions commemorative of his untimely decease! Truly an inscrutable Providence regulates the events of this life! Nothing but what pertains to the present moment is secure!

*Livingston County Medical Society.*—At a meeting of the Livingston County Medical Society, held in Geneseo, on the 18th inst., the President of the Society announced the deaths of W. C. Dwight, and George O. J. Du Relle. Whereupon Dr. W. C. Butler, D. H. Bissell, and L. J. Ames were appointed a committee to report suitable resolutions.

The following preamble and resolutions were reported and unanimously adopted:

Whereas, this society has lost by death, two of its prominent members, since its last meeting, viz., Wm. C. Dwight and G. O. J. Du Relle, who for several years past have been actively engaged in the practice of their profession, who were respected and beloved by their medical brethren, as well for their private virtues as for their distinguished ability—are cut down suddenly in the midst of their usefulness: and whereas, this society feel to deplore the loss of the worthy and esteemed members of the profession; therefore,

*Resolved,* That we deeply sympathize with the families of the deceased, and hereby offer them our heartfelt condolence.

*Resolved,* That a copy of the above resolutions be transmitted to the families of the deceased through the secretary of the society.

*Resolved,* That the above resolutions be published in the several newspapers of this county, and also in the Buffalo Medical Journal.

A. H. HOFF, President.

B. F. FOWLER, Secretary.

June 18, 1853.

